# Breaking the stalemate: a case of a laser-accelerated healing of a chronic tendon-exposed diabetic foot ulcer

**DOI:** 10.1093/jscr/rjag109

**Published:** 2026-06-19

**Authors:** Mohamed Hamdy Serour, Hussain Mohammad, Omar Aziza, Ibrahim Samir, AbdulAziz Almaheed, Naser Al-Humaidi

**Affiliations:** Department of General Surgery, Farwaniya Hospital, PO Box 13373, Kuwait City, Kuwait; Department of Orthopaedic Surgery, Jaber AlAhmad Hospital, PO Box 2270, Kuwait City, Kuwait; Department of General Surgery, Farwaniya Hospital, PO Box 13373, Kuwait City, Kuwait; Department of General Surgery, Farwaniya Hospital, PO Box 13373, Kuwait City, Kuwait; Department of General Surgery, Farwaniya Hospital, PO Box 13373, Kuwait City, Kuwait; Department of General Surgery, Farwaniya Hospital, PO Box 13373, Kuwait City, Kuwait

**Keywords:** diabetic foot ulcer, chronic wound, exposed tendon, adjunctive laser therapy, Erbium:YAG laser, case report

## Abstract

Chronic diabetic foot ulcers remain a major clinical challenge, particularly when complicated by tendon exposure and failure to respond to standard care. We report a 47-year-old male with Type II diabetes mellitus with a history of smoking who developed a chronic ulcer of the fourth web space of the left foot. Following resolution of an initial soft tissue infection, the wound progressed to a non-healing ulcer with exposed but viable extensor digitorum longus tendon. Despite guideline-directed management, healing plateaued. Adjunctive Erbium:YAG laser therapy was introduced only after failure of prolonged standard conservative management as a secondary intervention, resulting in progressive wound contraction, tendon coverage, and complete epithelialization by 8 weeks. This case illustrates a stepwise approach to chronic diabetic foot ulcer management and the potential role of laser therapy after failure of standard care.

## Introduction

Chronic diabetic foot ulcers are associated with prolonged healing and a high risk of complications despite guideline-directed management [1–3]. A subset of ulcers fails to progress with standard care, necessitating reassessment and escalation of therapy [3–6]. Tendon exposure further increases wound complexity and limits treatment options [7]. This report presents a chronic diabetic foot ulcer with preserved exposed tendon that failed to improve with standard management and subsequently demonstrated healing following the introduction of adjunctive laser therapy [8–10].

## Case presentation

**Figure 1:**
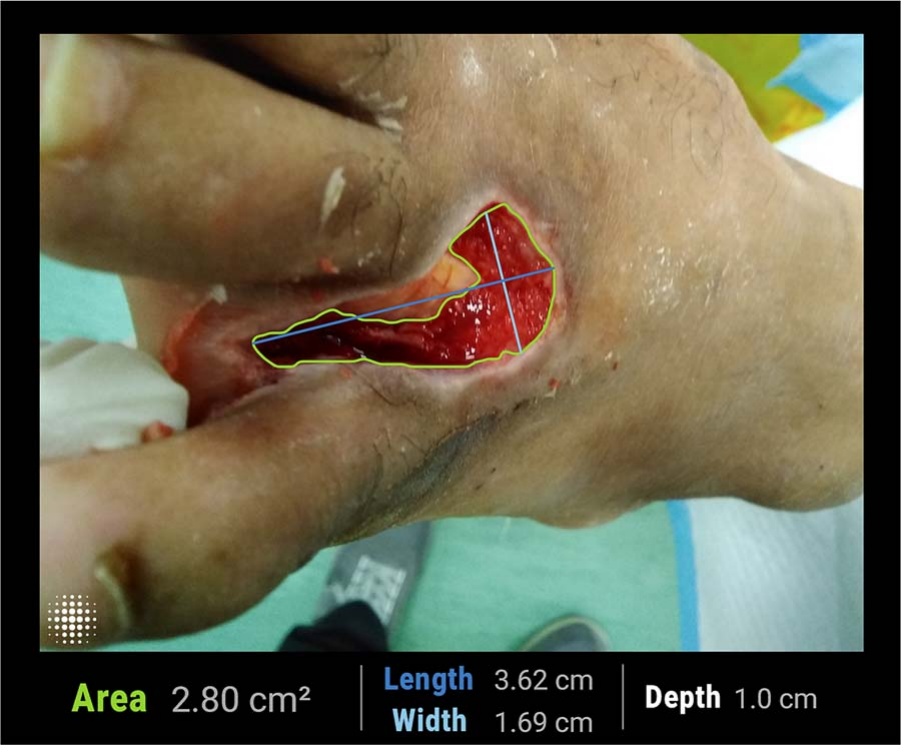
Clinical photograph of a chronic diabetic foot ulcer involving the fourth web space of the left foot at initiation of adjunctive laser therapy, demonstrating a deep ulcer with an exposed extensor digitorum longus tendon and a measured surface area of 2.8 cm^2^.

**Figure 2:**
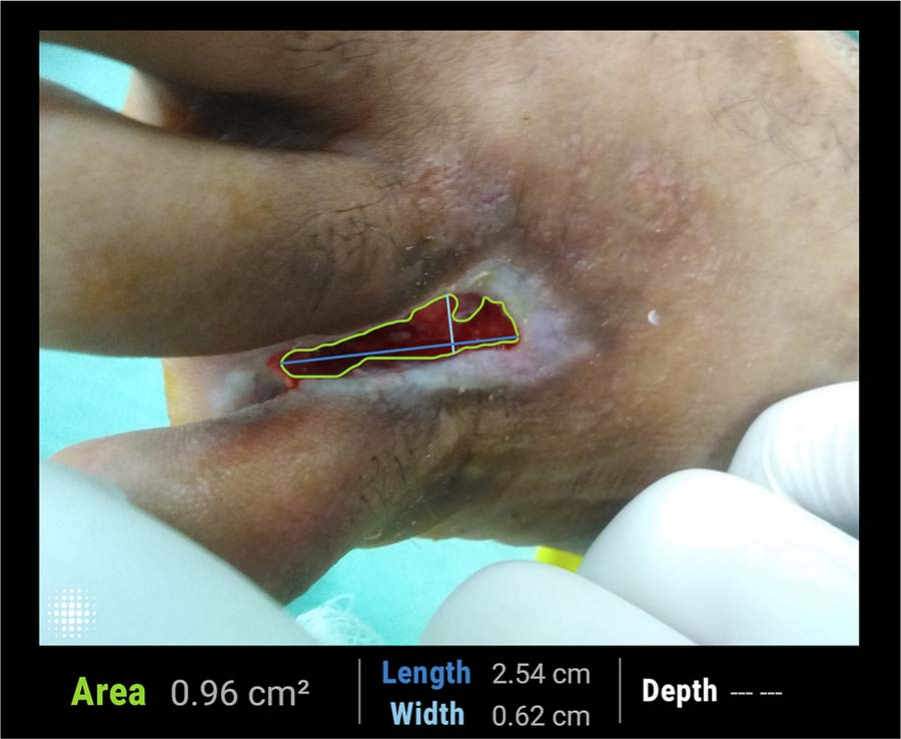
Clinical photograph after 3 weeks of adjunctive laser therapy, showing a reduction in ulcer surface area to 0.96 cm^2^ with early granulation tissue formation beginning to extend over the previously exposed tendon.

**Figure 3:**
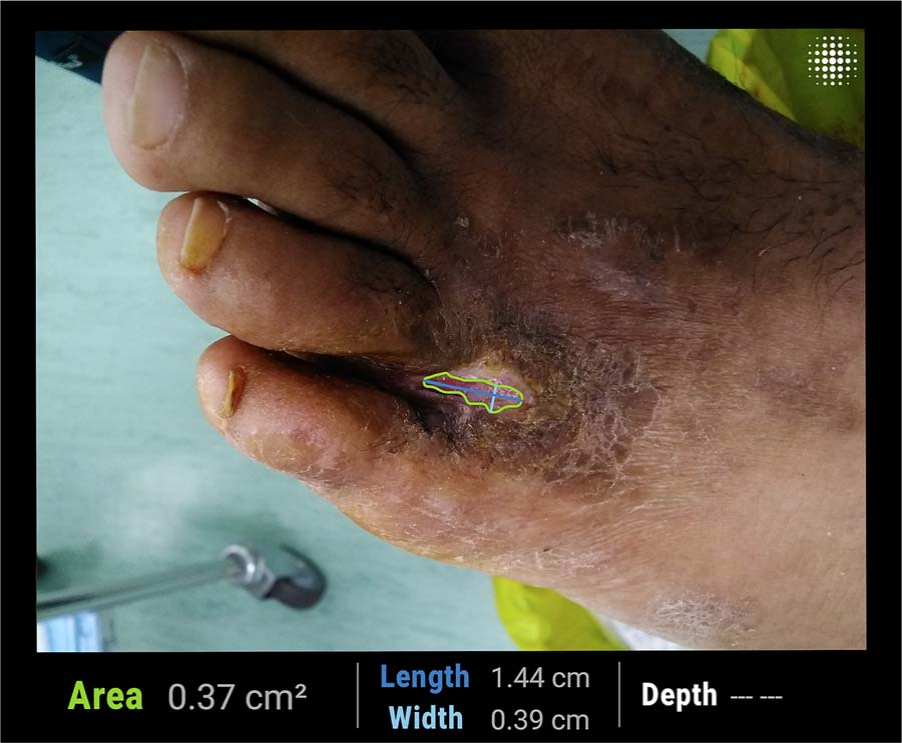
Clinical photograph after 6 weeks of adjunctive laser therapy, demonstrating further reduction in ulcer surface area to 0.37 cm^2^, complete coverage of the extensor tendon by granulation tissue, and a healthy wound bed.

**Figure 4:**
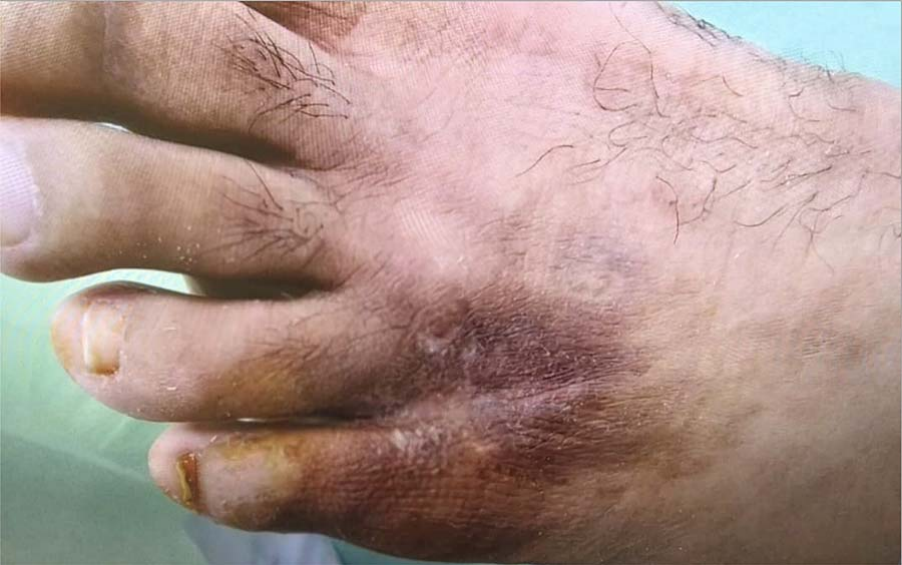
Clinical photograph at 8-week follow-up following initiation of adjunctive laser therapy, demonstrating complete epithelialization of the ulcer with no residual open wound.

A 47-year-old male with a background of Type II diabetes mellitus and a history of smoking presented with a chronic ulcer involving the fourth web space of the left foot. Clinical examination demonstrated intact pedal pulses and preserved distal perfusion, with no evidence of critical limb ischemia. Plain radiographs showed no features of underlying osteomyelitis. The ulcer initially developed following a localized soft tissue infection and was managed with incision and debridement performed on the day of admission, followed by minor surgical debridement and appropriate antibiotic therapy. Intravenous antibiotics were initiated and subsequently transitioned to culture-directed oral antibiotics after tissue cultures grew mixed gram-negative organisms sensitive to the prescribed regimen. Clinical improvement was observed, and the patient was discharged following completion of inpatient treatment. After resolution of the acute infection, the wound evolved into a chronic non-healing ulcer, characterized by a clean wound bed with persistent exposure of the extensor digitorum longus tendon, which was intentionally preserved and not excised due to its viable appearance and intact function. Subsequent management was conducted in accordance with the International Working Group on the Diabetic Foot recommendations, including infection control, surgical debridement, assessment of perfusion, appropriate offloading, and maintenance of a moist wound environment with regular reassessment. Standard conservative wound care was continued for 6 weeks; however, the ulcer demonstrated limited granulation response and wound healing progression plateaued with persistent tendon exposure. Other adjunctive wound therapies were considered. Hyperbaric oxygen therapy was not available at the treating institution. Negative pressure wound therapy was considered but was not pursued due to cost constraints and limited effectiveness in prior institutional experience for wounds with exposed tendon. The patient was reviewed by the plastic surgery team, who recommended either continued conservative management or excision of the exposed tendon in the absence of wound progression; therefore, a trial of adjunctive laser therapy was undertaken as a tendon-preserving measure prior to proceeding with tendon excision. Treatment was delivered as one session per week for a total of six sessions, in combination with continued standard wound care. At initiation of laser therapy, the ulcer measured 2.8 cm^2^ (Fig. 1). After 3 weeks, the wound area reduced to 0.96 cm^2^ (Fig. 2), with granulation tissue beginning to cover the exposed tendon. By 6 weeks, the ulcer had further reduced to 0.37 cm^2^ (Fig. 3), with complete tendon coverage and a healthy granulating wound bed. At 8-week follow-up, the wound achieved full epithelialization without complications (Fig. 4).

The patient was followed weekly by a diabetes specialist during the treatment course. Glycemic control was monitored using serial random blood glucose measurements, with a mean weekly value of approximately 6.5 mmol/l during the wound-healing period. The patient was a former smoker with a reported history of smoking seven cigarettes per day, with no change in smoking habits during the course of treatment.

## Discussion

Chronic diabetic foot ulcers represent a complex clinical problem associated with prolonged healing times and a high risk of complications, even when managed according to established care pathways [1–3]. In the present case, the ulcer persisted following resolution of acute infection and evolved into a chronic, non-healing wound with exposed extensor tendon despite adherence to guideline-directed management. This clinical course reflects previously reported observations that a proportion of diabetic foot ulcers fail to demonstrate meaningful progression despite appropriate standard-of-care measures [3].

Tendon exposure is a recognized marker of wound complexity and has been associated with delayed healing and increased risk of adverse outcomes [7]. In prior reports, exposed tendons in diabetic foot ulcers have frequently necessitated advanced interventions to achieve coverage and promote granulation [7]. In the current case, the extensor digitorum longus tendon was intentionally preserved due to its viable appearance and intact function, consistent with limb-preserving approaches described in the literature [7]. Despite this conservative strategy and ongoing standard wound care, the ulcer demonstrated limited granulation response and reached a clinical plateau.

Adjunctive laser therapy was not employed as an initial intervention. Management initially followed guideline-directed standard care, including infection control, surgical debridement, offloading, and maintenance of a moist wound environment with regular reassessment [4–6]. Despite these measures, the ulcer progressed to a chronic non-healing state with persistent tendon exposure, prompting reconsideration of the treatment strategy [3].

Objective wound assessment plays a critical role in identifying ulcers unlikely to heal with continued conservative management. Early change in wound area has been validated as a robust predictor of subsequent healing and as a practical decision point for treatment escalation [8]. In this case, the absence of meaningful wound contraction prior to laser therapy supported escalation rather than prolonged continuation of unchanged standard care.

Following the introduction of Erbium:YAG laser therapy, progressive wound area reduction and granulation tissue formation were observed. This temporal pattern is consistent with findings reported in clinical studies evaluating laser-based interventions in chronic ulcers refractory to standard treatment [9, 10]. Randomized and observational data have demonstrated that Erbium:YAG laser therapy can be associated with accelerated granulation and epithelialization when compared with conventional sharp debridement in selected chronic wounds [10, 11]. However, given the single-case design and the continued use of concurrent standard wound care measures, these observations should be interpreted as descriptive rather than causal, without attributing wound closure solely to laser therapy.

Other adjunctive modalities, such as negative pressure wound therapy, have also been reported to facilitate granulation and wound bed preparation in complex diabetic foot ulcers, particularly after surgical debridement [12, 13]. These studies collectively emphasize that adjunctive therapies may have a role when standard care fails to advance healing, rather than serving as first-line interventions. The present case aligns with this stepwise approach, illustrating escalation only after documented treatment refractoriness.

Although long-term glycemic control was not assessed, short-term glycemic status during wound healing was actively monitored and remained stable throughout the treatment period.

Overall, this case supports existing evidence that chronic diabetic foot ulcers with exposed tendon may fail to progress despite adherence to guideline-directed care and that structured reassessment can justify escalation to adjunctive therapies when healing plateaus [4, 5, 8].

## Conclusion

This case highlights the importance of structured reassessment in the management of chronic diabetic foot ulcers that fail to progress despite guideline-directed standard care. Ulcers complicated by exposed but viable tendon represent a challenging subgroup in which limb- and tissue-preserving strategies may be appropriate. When healing plateaus, escalation to adjunctive therapies can be considered. In this case, adjunctive laser therapy was introduced only after failure of standard measures and was associated with progressive wound closure, supporting its role as a secondary intervention in selected non-healing wounds.
